# Effect of Graph Scale on Risky Choice: Evidence from Preference and Process in Decision-Making

**DOI:** 10.1371/journal.pone.0146914

**Published:** 2016-01-15

**Authors:** Yan Sun, Shu Li, Nicolao Bonini, Yang Liu

**Affiliations:** 1 Key Laboratory of Behavioral Science, Institute of Psychology, Chinese Academy of Sciences, Beijing, China; 2 Department of Economics and Management, University of Trento, Trento, Italy; 3 College of Humanities & Social Sciences, University of Chinese Academy of Sciences, Beijing, China; Politecnico di Torino, ITALY

## Abstract

We investigate the effect of graph scale on risky choices. By (de)compressing the scale, we manipulate the relative physical distance between options on a given attribute in a coordinate graphical context. In Experiment 1, the risky choice changes as a function of the scale in the graph. In Experiment 2, we show that the type of graph scale also affects decision times. In Experiment 3, we examine the graph scale effect by using real money among students who have taken statistics courses. Consequently, the scale effects still appear even when we control the variations in calculation ability and increase the gravity with which participants view the consequence of their decisions. This finding is inconsistent with descriptive invariance of preference. The theoretical implications and practical applications of the findings are discussed.

## Introduction

Previous researchers have proposed that a common representation of magnitude or quantity exists for numerical and physical distances [1]. In line with such representational commonality, other researchers have found that the representation of numbers can be spatially organized along a mental number line, which is a continuum that is logarithmically compressed toward the right end as numbers increase in magnitude toward the right (see for example, [2–3]). As a result, the orientation of the mental number line facilitates (i.e., shorter response time) left-side responses to small numbers and right-side responses to large numbers [4].

Hevia et al. (2008) find that when people mentally conceive of extending a space, they are influenced by the magnitude of the numbers they are presented with, even when the numbers are irrelevant to the task [5]. This result suggests that information on magnitude modulates people’s conceptions of space, and that their mental visualization of space is involved in their mental conceptions about the relative size of numbers.

In a consumer context, Coulter and Norberg (2009) report a discount–distance congruency effect, which indicates that the perception of numerical difference between a regular and a discount price may be influenced by the physical distance between them. The authors find that a greater physical distance between the two prices can lead to a greater price discount perception, thereby increasing the likelihood of purchase [6].

Furthermore, Fias et al. (2003) demonstrate that a common cerebral representation of quantity is required for processing various forms of quantitative information [7]. Some studies have found that this common processing substrate specifically occurs in the intraparietal sulcus (see for example, [8]).

With the representational commonality between numerical distances and physical distances in mind, we look into the connection between graph transformation and decision making. Graphs are often used to present information when decisions are being made. Most people accept the idea that presenting data in the form of graphs or diagrams enhances data comprehension. However, personal decision making can sometimes be more easily biased when information is presented as graphs rather than as text [9–11]. Evidence shows that the physical properties of graphs influence an audience’s quantitative information perception (see for example, [12]).

**Fig 1:**
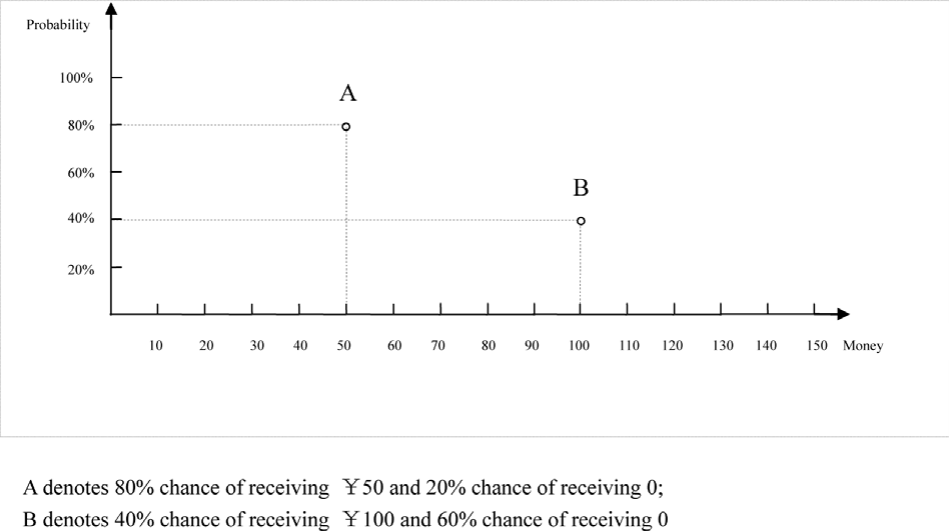
Probability distance compressed version.

**Fig 2:**
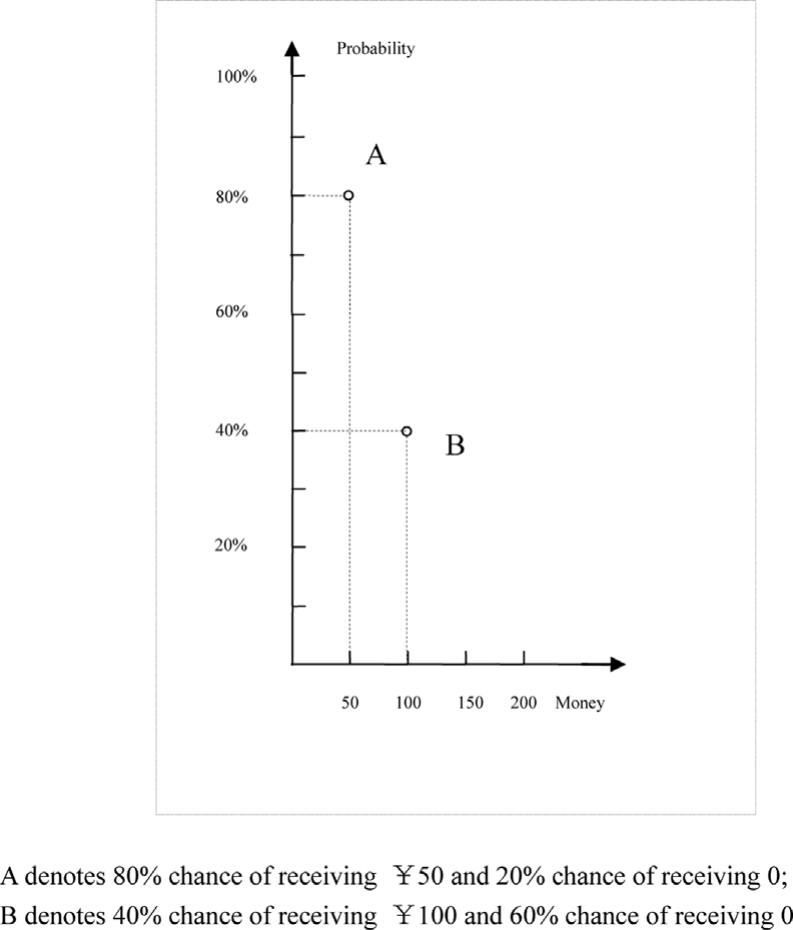
Money distance compressed version.

In this article, we investigate a specific physical property (i.e., physical distance) on coordinate graphs. The relative physical distance between options on a coordinate graph is usually formed by the objective numeric values of relevant attributes. However, the physical distance on a graph may also change as a function of the scale employed in the graph, even when the same numeric values are presented. In a binary risk choice, for example, the physical distance between options A and B is longer on the X-axis when the choice is presented as in Fig 1 than when it is presented as in Fig 2; the distance on the Y-axis is exactly the opposite.

According to the representational commonality of numerical and physical distances, magnitude representations related to both relevant (i.e., numerical difference) and non-relevant (i.e., physical distance) stimulus dimensions should be automatically evoked upon exposure to a coordinate graph. Thus, the physical distances in Figs 1 and 2 are expected to affect the corresponding perception of the numerical differences. That is, using different scales on graphs can result in a perception of a higher (lower) numerical difference between options A and B with respect to the probability (money) attribute in Fig 2 than in Fig 1. Therefore, the numerical difference between 80% and 40% would be greater in Fig 2 than in Fig 1. The opposite would be true for a viewer’s perception of the numerical difference between ¥50 and ¥100.

In a choice situation, such a perception of differences in attributes can often be a preference determinant, according to attribute-based choice models. For example, the similarity choice model indicates that individuals base their decisions on judgments about the similarity or dissimilarity of attributes across alternatives [13–17]. If two options appear similar with respect to a given decision attribute (i.e., prize or probability), that attribute is assigned little or no weight in a choice. Accordingly, if we use different figures (e.g., Figs 1 and 2) to present information about choices, preference could change as a function of the similarity between attributes, as predicted by the similarity choice model (in this case, the closer the options are on an axis, the more similar they appear and the less likely a viewer mentally assigns a difference between them).

We therefore hypothesize that manipulating a viewer’s perception of the distance between options by (de)compressing graph scale will directly affect the viewer’s preference, even when the physical distance on the graph is a task-irrelevant feature. We conduct experiments to test this “distance-as-a-cue for preference” hypothesis.

## Experiment 1

In Experiment 1, we varied the relative distance between options on a given attribute by (de)compressing the scale in a graph to test whether such a change can affect risky choices. Specifically, we expect the proportion of viewers selecting option A to increase from Figs 1 to 2.

### Method

#### Ethics Statement

This study was reviewed and approved by the Ethics Committee of the Institute of Psychology, Chinese Academy of Sciences. Written consent was also obtained from all the participants before the experiment according to the established guidelines of the committee. This procedure was followed in Experiments 2 and 3 as well.

#### Participants

Posters were displayed in schools to recruit participants. We began the study with an initial pool of 192 undergraduate student volunteers, who provided oral consent. All the participants were given a small gift for their participation. Three incomplete questionnaires were excluded from the analysis.

#### Materials and procedure

The participants were randomly assigned to one of the two graph-scale conditions depicted in Figs 1 and 2 (i.e., compressed probability distance and compressed money distance). They were asked to choose one of the two options from each choice pair. In each choice pair, one of the options is called the “probability bet” because it has a higher probability of winning, whereas the other option is termed the “money bet” because the amount of winnings is higher in this choice. In the following pair of bets, for example, option A is the probability bet and option B is the money bet. The expected value for an option in risky choices is expressed as the sum of all the products of the outcomes multiplied by their respective probabilities. Note that the expected values of option A and option B are the same.

*A*. *80% chance of winning $50*, *20% chance of winning $0 (*probability bet)

*B*. *40% chance of winning $100*, *60% chance of winning $0 (*money bet)

Each of the 189 participants was presented with four pairs of bets (Table 1), yielding 756 observations. About half of the participants answered four questions in the order given in Table 1, whereas the others answered the same questions in reverse order. The dependent variable is the percentage of the probability bets in each choice pair.

**Table 1:** The percentages of the probability-bet in choices between the graph-scale conditions in Experiment 1.

Pairs		Scale Condition	
		Probability distance compressed version (N = 95)	Money distance compressed version (N = 94)
1.	A:80%,¥50	62%	73%
	B:40%,¥100		
2.	A:60%,¥100	41%	60%
	B:40%,¥150		
3.	A:60%,¥50	55%	70%
	B:20%,¥150		
4.	A:40%,¥50	44%	68%
	B:20%,¥100		

### Results and Discussion

The percentage of the probability bets in each choice pair (Table 1) is consistent with our prediction. To illustrate, in Pair 1, 62% of the participants prefer the probability bet in the compressed probability distance condition. However, the choice percentage increases to 73% in the compressed money distance condition.

The results of the four choice pairs are tested separately using *χ*^2^ test, which confirmed our prediction that the percentage of the probability bets in each choice pair increased from the probability distance compressed version to the money distance compressed version. Specifically, the differences were statistically significant in three choice pairs: *χ*_2_^2^ (1, 189) = 6.484, *p* < .01 for choice pair two; *χ*_3_^2^ (1,189) = 4.825, *p* < .05 for choice pair three; *χ*_4_^2^ (1,189) = 9.029, *p* < .01 for choice pair four, and marginally significant in choice pair one: *χ*_1_^2^ (1,189) = 2.760, *p* = .066. In addition, a comprehensive analysis on the overall experiment results is to run a log linear analysis with a full model (i.e., condition, item and condition by item).

The results of Experiment 1 confirm the “distance-as-a-cue for preference” hypothesis, contrary to descriptive invariance of preference, which requires that logically equivalent ways of describing choice options yield the same preference order. The results also show that changing the relative distance of options on a given attribute by graph scale (de)compression affects preferences.

## Experiment 2

In Experiment 1, risky choice changes as a function of the scale in the graph. In Experiment 2, we test whether graph scale also affects features of the decision process, such as decision time.

It is well known that, rationally, people should choose the option with higher expected value in risky choices [18,19]. Obviously, the expected value for an option will vary as a function of the dimensions (i.e. outcome and probability) while a choice is made. However, we believe that the scale manipulation effect we observed should be independent of the parameters of the choices. Consider the following two pairs of bets:

*Pair 1 [Dominance of money bet]*:

*A*. *80% chance of winning $50 B*. *70% chance of winning $100*

*Pair 2 [Dominance of probability bet]*:

*A*. *60% chance of winning $50 B*. *20% chance of winning $60*

The expected value is higher for the money bet (i.e., option B) in Pair 1 and for the probability bet (i.e., option A) in Pair 2. For both pairs, however, the physical distances between the options in the compressed money distance condition always favor option A.

Logically, if linear spatial distances play a role in graphical decision making, then decision times should be shorter when the physical distance and expected value support the same option; decision times should be longer when they support different options. The decision times in Pair 2 are expected to be shorter in the compressed money distance condition than in the compressed probability distance condition. This is because the option that the spatial distance favors is the same one that the expected value favors in the first figure condition, but this is not the case in the second figure condition. The opposite would be true in Pair 1, as illustrated in Table 2.

**Table 2:** Changes in the favored option as a function of figure condition and expected value.

Expected value	Figure condition	
	Probability distance compressed version (favors option B)	Money distance compressed version (favors option A)
**Pair 1**		
**(in favor of B)**	**congruent**	**incongruent**
**Pair 2**		
**(in favor of A)**	**incongruent**	**congruent**

In Experiment 2, we designed two kinds of choices in accordance with the expected value of the bets (i.e., *money advantage vs*. *probability advantage*) and presented them in two figure conditions (e.g., *compressed money distance vs*. *compressed probability distance*). The probability bet has a higher (lower) expected value than the monetary-bet in the probability (money) advantage choices; thus, we predict an interaction between choice category and figure condition. That is, the response time for the probability advantage choices are expected to be shorter in the compressed money distance option than in the compressed probability distance condition, but the reverse is expected for the money advantage choices.

In addition to decision time, changes in preference were also investigated. We want to check whether the scale effect in preferences would also occur when each of the bets in the pair has different expected values. The probability bet has the same expected value as the monetary bet in Experiment 1, but this is not true in Experiment 2. If people simply rely on expected values in making choices and ignore spatial distance, then no scale effects will be observed in Experiment 2. People are expected to systematically prefer the bet with the higher expected value. Considering the findings in Experiment 1, however, we expect to find a scale effect in their preferences. Specifically, the probability bet would be chosen more frequently in the compressed money distance condition than in the compressed probability distance condition, and vice versa.

### Pilot Experiment

The goal of the pilot experiment is to examine the effect of choice parameters on preferences. We determined whether the participants favor the probability bet in the probability advantage choices but prefer the money bet in the money advantage choices. A separate sample of 16 participants indicated their preferences on 18 pairs of bets, which were presented in numerical format. Higher expected values were attached to the money bet for 9 money advantage choices, and higher expected values were assigned to the probability bet for the other 9 probability advantage choices. In keeping with the design of the experiment, the data show that most of the participants prefer the probability bet in the probability advantage choices but prefer the money bet in the money advantage choices (binomial test, *p* < .01 for all the choices).

The goal of the main experiment is to demonstrate the effect of graph scales on decision making from two perspectives. For decision-making output, we predict a significant effect of graph scales on decision-making preferences, as observed in Experiment 1. For decision-making processes (i.e., decision time), we predict a significant interaction between choice category and figure condition.

### Method

#### Participants

Forty-four undergraduate students participated in Experiment 2. Posters were displayed in campuses to recruit the participants, who were paid ¥20 (approximately US$3.10 at the time) for joining the study. The experiment was conducted in groups of six participants. One participant was excluded from the analyses because he violated the criterion of preference dominance with a filler item.

#### Materials and procedure

The same 18 pairs of bets that were used in the pilot experiment were presented to each participant in graphical format. Each choice included two different versions (e.g., compressed probability distance vs. compressed money distance), as in Experiment 1. Participants were randomly assigned to each version: 23 participants were asked to respond to the compressed probability distance condition, while the remaining 21 were asked to respond to the compressed money distance condition. The order of presentation of the 18 pairs was randomized. Thus, this experiment is a 2 (choice category: probability advantage vs. money advantage) × 2 (figure condition: compressed money distance vs. compressed probability distance) mixed experimental design, with figure condition as the between-subjects factor and choice category as the within-subjects factor.

The experiment was conducted with the aid of a computerized procedure. A description of the ostensible purpose of the study was provided before the experiment, along with instructions for the task. The participants were told that the study was designed to investigate preferences in risk choices, and that they should choose one bet as quickly as possible for each pair.

To eliminate incorrect results caused by subject inattention, we presented each participant with a filler choice: (A) receive ¥110 with a probability of 0.5 and (B) receive ¥100 with a probability of 0.4. If a participant prefers B to A, his/her response is regarded as a violation of dominance because option B is dominated by A in this choice. As a result, the data from one participant were excluded.

### Results and Discussion

#### Response time results

**Fig 3:**
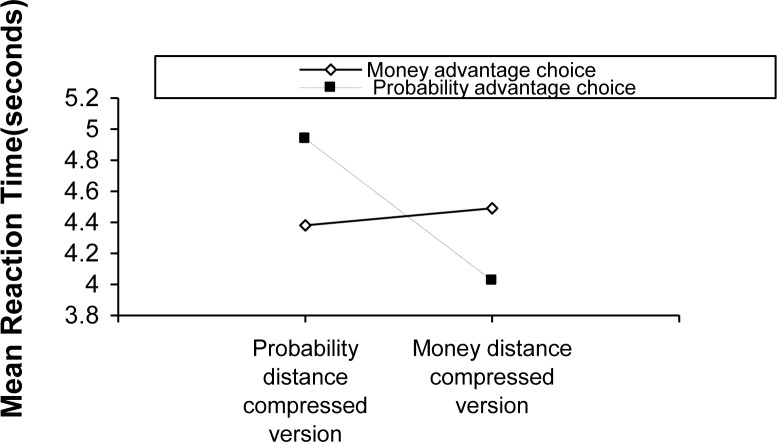
Mean response time as a function of choice category and figure condition in Experiment 2.

The mean response times as a function of choice category and figure condition are shown in Fig 3.

The figure shows that the response time for probability advantage choices is shorter in the compressed money distance condition (Mean = 4.024 seconds, Standard Deviation = 1.306 seconds) than in the compressed probability distance condition (M = 4.938 seconds, SD = 1.878 seconds). However, the response time for money advantage choices is longer in the compressed money distance condition (M = 4.490 seconds, SD = 1.461 seconds) than in the compressed probability distance condition (M = 4.381 seconds, SD = 1.912 seconds). A 2×2 ANOVA on response time reveals an interaction between choice category and figure condition (F(1, 41) = 6.482, *p* < .05). However, the main effects are non-significant for either choice category (F(1, 41) = 0.051, *p* >.05) or figure condition (F(1, 41) = 0.724, *p* >.05), indicating that response time is not sensitive to choice category or figure condition alone.

The most relevant result for response time is the interaction between choice category and figure condition. As predicted, participants responded more rapidly when physical distance (i.e., figure condition) and expected values (i.e., choice category) were congruent than when they were incongruent. This finding suggests that physical distance plays a role in decision-making processes. In sum, the data on response time support the hypothesis that changing the relative distance of options on a given attribute by (de)compressing graph scale can affect decision making, even without a change in objective values.

#### Choice results

We computed the percentage of choosing the probability bet for each participant in both the probability advantage choice condition and the probability advantage choice condition. The percentages of probability bet as a function of choice category and figure condition are shown in Table 3.

**Table 3:** The mean choice percentages of the probability bet from the choices in Experiment 2.

Choice category	Figure condition	
	Probability distance compressed version (N = 23)	Money distance compressed version (N = 20)
**Money advantage choices**	**6%**	**24%**
**Probability advantage choices**	**86%**	**87%**

As expected, the scale effect is also found in the choice results of Experiment 2. A 2×2 ANOVA on the percentage of probability bet reveals the main effect for figure condition (F (1, 41) = 6.073, *p* <. 05), indicating that the probability bet is chosen more frequently in the compressed money distance condition than in the compressed probability distance condition. Consistent with the results of the pilot experiment, the main effect of choice category is also significant (F (1, 41) = 278.519, *p* <. 001), showing most of the participants prefer the probability bet in the probability advantage choices, but not in the money advantage choices. The effect of the interaction between choice category and figure condition is also significant (F (1, 41) = 4.435, *p* <. 05).

It could be argued that the ANOVA above could not satisfy the normality assumption, so a nonparametric test was run to test the scale effect. In the category of money advantage choices, the probability bet is chosen significantly more frequently in the compressed money distance condition than in the compressed probability distance condition, Mann-Whitney U test, U = 125, *p* < .01. In the category of probability advantage choices, no significant difference was found on preference for probability bet in the compressed money distance condition and in the compressed probability distance condition, Mann-Whitney U test, U = 125, *p* = .53. These results partly supported the scale effect.

A participant’s preference between simple gambling options is a function of expected values. The main effect of choice category is considerably stronger than that of figure condition. The expected values weigh more heavily than do relative distances between options in the decision-making process. However, the transformation of scale still affects preference. The results of the participants’ choices also demonstrate that people use the perceived distance between bets on a given attribute as a cue for making choices, even in contexts where the bets have different expected values.

## Experiment 3

Experiments 1 and 2 demonstrate the effect of graph scales on decision making, but several potential problems still require consideration. First, we failed to control the statistics knowledge base of the participants in the first two experiments. Consequently, the decisions of students who have taken statistics courses and calculated the expected value may differ significantly from those of other students. Second, because hypothetical rather than actual choices were used in Experiments 1 and 2, the effect of graph scales on choice could drastically decrease and may become negligible if the decision makers view the consequences of their decisions in a serious manner. We resolve these issues in Experiment 3.

### Method

#### Participants

The initial participant pool consisted of 142 undergraduate student volunteers. All the participants were given a small gift for their participation. To control the variations in the statistics knowledge base of the decision makers, we recruited only the participants who had completed statistics courses. Two incomplete questionnaires were excluded from the analysis.

#### Materials and procedure

The first choice pair from Experiment 1 (Pair 1, Table 1) was presented to all the participants in graphical format. We used only the one-shot pair of bets because the participants were experts in statistics, and the presentation of multiple bets with different expected values might remind them to use the learned expected value in opting for the risky choice. The participants were randomly assigned to each questionnaire version: 67 participants were asked to respond to the compressed probability distance condition (i.e., Fig 1), and the remaining 73 were asked to respond to the compressed money distance condition (i.e., Fig 2). They were asked to choose one of two bets. To encourage the participants to ponder over the consequences of their decisions, we told them that six of them would be randomly selected to play the actual game that they had chosen in the questionnaire. That is, they would be paid real money according to the consequences of their bets.

### Results and Discussion

The choice results are consistent with the pattern in Experiment 1. Specifically, 57% of the participants prefer the probability bet (i.e., 80% chance of winning ¥50) over the money bet (i.e., 40% chance of winning ¥100) in the compressed probability distance condition. However, the choice percentage for the probability bet increases to 74% in the compressed money distance condition. The difference between the two graph versions is statistically significant (χ^2^ (1) = 4.617, *p* < .05), indicating a significant effect of graph scales on decision-making preferences.

In Experiment 3, we examined the scale effect in a more rigorous manner. First, only the participants who had completed statistics courses were recruited. This approach eliminates the possibility that the scale effects appear merely because the decision makers are unable to calculate the expected values. Second, the actual money incentive was employed as a way to encourage the participants to view the consequence of their decisions with concern. Thus, the scale effects would not have resulted from the absence of concern over the experiment.

## General Discussion

Although in the literature many researchers argued on the role of graph presentation on judgment and choice, we proved it by a set of coordinated experimental studies. Specifically, we investigated the effect of graph scale on risky choices, in which we manipulated the relative physical distance between options on a given attribute by (de)compressing graph scale. Such manipulation affects decision making in a coordinate graphical context. Experiment 1 shows that risky choice changes as a function of graph scale, while Experiment 2 demonstrates that the type of graph scale also affects decision times. The participants responded more rapidly when under congruent rather than incongruent conditions. Experiment 3 features a more rigorous examination of the graph scale effect. The scale effects still appeared even when we controlled the variations in calculation ability and increased the gravity with which the participants viewed the consequence of their decisions. These experiments indicate that physical distance on a coordinate graph influences decision making.

This work contributes to the literature in four aspects. First, although previous research has shown that different representations of the same choice problem do not yield the same preference [20–25], we validate this finding in a graphical rather than a verbal context. Our findings therefore challenge descriptive invariance of preference without manipulating the verbal and numerical description of problems, and extend previous knowledge about the dependence of preferences on problem formulation.

Second, although several studies have shown that changes in the perception of weights/values and probabilities can be obtained by the manipulation of the aggregation level of the attributes and events to be judged [26–27], in our study the aggregation level was the same between the two versions of the problem. To this aspect, our findings add a new factor (scale compression) as a potential way to alter perception of value of attributes and related preferences in a binary choice context.

Third, we provide evidence for partial interoperability and interchangeability between numerical and physical distances from a new prospective. Previous studies primarily tested the mutual influence between the perceptions of numerical and physical distances to support representational commonality. By contrast, our findings demonstrate the reasonability of this conjecture by showing the effect of physical distances on risky choices that should be determined only by numerical distances. The current study therefore expands our understanding of the fundamental reciprocity that occurs between perceptions of numerical and physical distances.

Fourth, beyond comparing preferences across graph versions, we also traced decision processes by measuring decision times. If people simply ignore spatial distance then no interaction should occur between choice category and figure condition in terms of decision time. This argument does not hold, however. The significant interaction between choice category and figure condition indicates that physical distance plays a role in decision-making processes. Thus, we have provided convergent evidence from both preference and decision processes for the effect of graph scales on decision making.

Our results can be understood at a broader theoretical level by relating them to the theory of natural assessment [28]. This theory maintains that physical properties such as size, distance, and loudness are evaluated routinely and automatically without effort. Hence, the evaluations of these physical properties are often substituted for more complicated judgments. Within the framework of substitution judgment, graph scale effects occur simply because evaluating physical distance is easier than calculating numerical difference. Therefore, the physical distance on a coordinate graph as a physical property is a partial substitute for the corresponding numerical difference in decision-making processes.

Practically speaking, perceived distance as a cue for preference may also influence consumer choice and managerial decision making. For example, Coulter and Norberg (2009) show that the more distant a printed discounted price is from the full price in an ad, the higher the likelihood that the commercial offer will be accepted [6]. This finding is of interest to marketing professionals and consumer law practitioners. The European Union has issued directives for promoting informed consumer decision making, and avoiding deceptive communication between firms and consumers (Directive 2005/29/CE of 11 May 2005; Directive 2006/114/CE of 12 December 2006). Our study shows that manipulation in a graph of perceived distance between risk options affects choice. Note that our study uses graphs to present the information of choices and thus exerts impact on the final choices, rather than uses statistic knowledge to mislead people to make uncertain decisions. In addition, the present result is generalizable to other risky consumer choice domains. Consider, for example, a pension plan or financial product in which the relevant attributes are returns and risks. In this context, products that offer higher returns are associated with higher risks. On the basis of our results, we conclude that a graph that compresses risk attributes will likely encourage risk-seeking behavior in consumers (e.g., choose the financial product that is associated with higher returns even if the risk is higher). On the other hand, one may wonder whether there are some methods which could be applied to counterbalance this distance-as-cue heuristic, such as judging the area when the coordinate axes both include zero, or judging the diagonal rather than distance. It is interesting and important to explore this issue in the future research in order to making better judgment and decisions.

This study has identified a scale manipulation effect on decision making in graphical contexts, indicating that graphs may be manipulated to elicit desired impressions of the same information. As inconsequential as this manipulation may appear, it effectively changes preferences. With the increasing popularity of all kinds of graphs in information communication, using graphical representations is worth considering when presenting information in any context that involves graphs.

## Supporting Information

S1 File Dataset: experiment 1. (SAV)

S2 File Dataset: experiment 2. (SAV)

S3 File Dataset: experiment 3. (SAV)
